# A comprehensive assessment of patient reported symptom burden, medical comorbidities, and functional well being in patients initiating direct acting antiviral therapy for chronic hepatitis C: Results from a large US multi-center observational study

**DOI:** 10.1371/journal.pone.0196908

**Published:** 2018-08-01

**Authors:** Donna M. Evon, Paul W. Stewart, Jipcy Amador, Marina Serper, Anna S. Lok, Richard K. Sterling, Souvik Sarkar, Carol E. Golin, Bryce B. Reeve, David R. Nelson, Nancy Reau, Joseph K. Lim, K. Rajender Reddy, Adrian M. Di Bisceglie, Michael W. Fried

**Affiliations:** 1 Division of Gastroenterology and Hepatology, Department of Medicine, University of North Carolina, Chapel Hill, North Carolina, United States of America; 2 Department of Biostatistics, University of North Carolina, Chapel Hill, North Carolina, United States of America; 3 Division of Gastroenterology and Hepatology, Department of Medicine, University of Pennsylvania, Philadelphia, Pennsylvania, United States of America; 4 Division of Gastroenterology and Hepatology, Department of Internal Medicine, University of Michigan, Ann Arbor, Michigan, United States of America; 5 Division of Gastroenterology, Hepatology & Nutrition, Department of Internal Medicine, Virginia Commonwealth University, Richmond, Virginia, United States of America; 6 Division of Gastroenterology and Hepatology, Department of Medicine, University of California Davis, Davis, California, United States of America; 7 Division of General Medicine and Clinical Epidemiology, Department of Medicine, School of Medicine, University of North Carolina, Chapel Hill, North Carolina, United States of America; 8 Department of Health Behaviors, Gillings School of Global Public Health, University of North Carolina, Chapel Hill, North Carolina, United States of America; 9 Department of Population Health Sciences, Duke University, Durham, North Carolina, United States of America; 10 Division of Gastroenterology, Hepatology & Nutrition, Department of Medicine, University of Florida, Gainesville, Florida, United States of America; 11 Department of Internal Medicine, Section of Hepatology, Rush University, Chicago, Illinois, United States of America; 12 Digestive Diseases, Department of Internal Medicine, Yale University, New Haven, Connecticut, United States of America; 13 Division of Gastroenterology and Hepatology, Department of Internal Medicine, Saint Louis University, St. Louis, Missouri, United States of America; National Taiwan University Hospital, TAIWAN

## Abstract

**Background:**

Symptom burden, medical comorbidities, and functional well-being of patients with chronic hepatitis C virus (HCV) initiating direct acting antiviral (DAA) therapy in real-world clinical settings are not known. We characterized these patient-reported outcomes (PROs) among HCV-infected patients and explored associations with sociodemographic, liver disease, and psychiatric/substance abuse variables.

**Methods and findings:**

PROP UP is a large US multicenter observational study that enrolled 1,600 patients with chronic HCV in 2016–2017. Data collected prior to initiating DAA therapy assessed the following PROs: number of medical comorbidities; neuropsychiatric, somatic, gastrointestinal symptoms (PROMIS surveys); overall symptom burden (Memorial Symptom Assessment Scale); and functional well-being (HCV-PRO). Candidate predictors included liver disease markers and patient-reported sociodemographic, psychiatric, and alcohol/drug use features. Predictive models were explored using a random selection of 700 participants; models were then validated with data from the remaining 900 participants. The cohort was 55% male, 39% non-white, 48% had cirrhosis (12% with advanced cirrhosis); 52% were disabled or unemployed; 63% were on public health insurance or uninsured; and over 40% had markers of psychiatric illness. The median number of medical comorbidities was 4 (range: 0–15), with sleep disorders, chronic pain, diabetes, joint pain and muscle aches being present in 20–50%. Fatigue, sleep disturbance, pain and neuropsychiatric symptoms were present in over 60% and gastrointestinal symptoms in 40–50%. In multivariable validation models, the strongest and most frequent predictors of worse PROs were disability, unemployment, and use of psychiatric medications, while liver markers generally were not.

**Conclusions:**

This large multi-center cohort study provides a comprehensive and contemporary assessment of the symptom burden and comorbid medical conditions in patients with HCV treated in real world settings. Pain, fatigue, and sleep disturbance were common and often severe. Sociodemographic and psychiatric markers were the most robust predictors of PROs. Future research that includes a rapidly changing population of HCV-infected individuals needs to evaluate how DAA therapy affects PROs and elucidate which symptoms resolve with viral eradication.

**Trial registration:**

(Clinicaltrial.gov: NCT02601820).

## Introduction

Chronic hepatitis C virus (HCV) infection affects over 3 million Americans and is a leading cause of liver failure, cirrhosis, and liver cancer[1, 2]. Though primarily associated with liver disease, recent evidence suggests that HCV is a multi-faceted systemic condition that may be linked to many extra-hepatic disorders (EHDs) including arthritic-like pain, endocrine, metabolic, kidney, neuropsychiatric, and cardiovascular conditions [3–5]. Studies conducted during the interferon (IFN) treatment era found associations between HCV and neuropsychiatric, somatic and gastrointestinal (GI) symptom clusters, most commonly fatigue, sleep disturbance, irritability, depression, and pain[6–11]. Symptoms may be attributable to liver disease or to underlying inflammatory processes that mediate the relationship between HCV infection and EHDs [4, 12]. Beyond disease-related factors, psychosocial factors may also contribute to symptom burden. Psychiatric and substance use disorders, salient risk factors for contracting HCV, are highly prevalent among HCV patients and may be directly associated with poor health outcomes, irrespective of HCV infection or liver disease[9, 10, 13–15]. Indeed, neuropsychiatric symptoms are reported in up to 50% of HCV patients, independent of liver disease severity[16]. Additionally, the HCV population is further disadvantaged by social determinants of poor health (SDoH), including economic instability, low income, unemployment and lack of health insurance[17–19]. Finally, the psychological sequelae of harboring a transmittable disease, social stigma, and anxiety related to deteriorating liver health may also contribute to symptom burden and poor health outcomes[20–24]. Fortunately, the impact of these factors on health outcomes may be mitigated by public awareness that a short course of well tolerated direct acting antiviral (DAA) therapy has over 95% efficacy in achieving virologic cure.

Much of the data on HCV-associated symptoms, health-related quality of life (HRQOL), and treatment side effects, comes from the IFN treatment era [6–8, 25]. *Qualitative* studies elucidated patients’ experiences of symptoms and HRQOL[7, 26–28], but far fewer *quantitative* studies provided any in-depth analysis of HCV symptoms, especially among patients not on IFN therapy[6, 7]. During the IFN era, researchers identified 22 key patient-reported outcome (PRO) concepts from qualitative studies as important to the HCV population[7]. The majority of these PRO concepts received inadequate attention then and have received virtually no attention during the current DAA era. Several recent studies of HCV-infected patients treated with DAAs have investigated a few PRO concepts, such as HRQOL, fatigue, and work productivity[29, 30]. However, these data were derived from drug registration trials that enrolled highly selected patients with limited participation of under-represented racial minorities and subgroups with decompensated liver disease, EHDs, and medical, psychiatric and addiction comorbidities.

A comprehensive understanding of baseline symptom burden in patients with HCV is necessary to lay the groundwork for subsequent real-world investigations of potential changes in symptoms during DAA therapy and after virologic cure. We aimed to characterize patient-reported symptoms, medical conditions, and functional well-being in a large multi-center US cohort who initiated DAA therapy in clinical practices in 2016-2017. Our secondary aim was to evaluate sociodemographic/SDoH, liver-related, and other clinical features associated with these health outcomes.

## Methods

### Study design

The Patient-Reported Outcomes Project of HCV-TARGET (PROP UP) study is funded by the Patient-Centered Outcomes Research Institute (PCORI) and is a unique HCV study developed with engagement of patients affected by HCV and patient advocates. PROP UP is a multi-center, prospective, observational study that enrolled 1,600 patients across the U.S. to characterize patients’ experiences associated with HCV, DAA treatments, and virologic cure. The rationale and study protocol for PROP UP has been previously published[31]. Site recruitment began in January 2016 and ended in October 2017 at 11 U.S. medical centers (9 academic hepatology and 2 private gastroenterology). All sites were under the jurisdiction of their local Institutional Review Board (IRB) and obtained approval prior to study initiation (S1 Table). Inclusion criteria were made purposely broad to capture real world clinical experiences, and mainly required completion of baseline PROs and initiation of one of five DAA regimens. The current analysis used cross-sectional data collected at baseline *prior to* patients starting DAA therapy.

### Measures

Brief details of the measures are provided below, as extensive details are provided elsewhere[31].

#### Number of medical comorbidities

Participants responded to a list of 30 chronic medical conditions regarding whether they (a) never had the condition; (b) have experienced the condition in the past; or (c) have experienced the condition in the last year (current condition). For these analyses we focused on predictors of current medical comorbidities.

#### Specific symptoms

The National Institutes of Health PROMIS instruments were used to capture three symptom clusters associated with HCV in the literature: neuropsychiatric (depression, anxiety, anger, cognitive concerns); somatic (pain interference, fatigue, sleep disturbance); and GI (abdominal pain, nausea/vomiting, diarrhea) clusters[32–34]. Psychometric testing of these PROMIS instruments in patients with HCV demonstrated satisfactory reliability and validity[35, 36]. Higher PROMIS T-scores reflect worse symptoms.

#### Overall symptom burden

A comprehensive list of 32 symptoms common to many medical conditions were assessed using the Memorial Symptom Assessment Scale (MSAS)[37, 38]. Participants reported the presence or absence of symptoms, and if present, its severity, frequency and level of distress. A total symptom burden (TMSAS) score was calculated. A higher TMSAS score reflects higher symptom burden.

#### Functional well-being

The HCV-PRO is a newly developed HCV-specific survey designed to evaluate the functional well-being of patients with HCV[39, 40]. The scale includes 16 items that measure various aspects of physical and emotional functioning, productivity, intimacy, and perceived quality of life related to having HCV. The total score ranges from 0 to 100; higher scores indicate better functional well-being.

#### Sociodemographics

Participants reported age, sex, racial background, educational attainment, annual household income, marital status, employment status, and health insurance status were explored as potential predictors of PROs.

#### Liver-related clinical and laboratory markers

The following data were extracted from participants’ medical records by trained site coordinators: HCV genotype, HCV RNA level, aspartate aminotransferase test (AST), alanine aminotransferase test (ALT), albumin, total bilirubin, platelets, hemoglobin, creatinine, and international normalized ratio (INR). Based on these laboratory data, we calculated various measures of advanced fibrosis/cirrhosis: (a) the AST to platelet ratio index (APRI); (b) the Fibrosis-4 Index for Liver Fibrosis (FIB-4), and (c) the Model for End-Stage Liver Disease (MELD), where FIB-4 >3.25 indicated advanced fibrosis; APRI >2.0 and MELD >6 indicated cirrhosis, and MELD ≥12 indicated advanced cirrhosis[41–43]. Site coordinators were also trained to review multiple sources of evidence in patient medical records, such as biopsy results, ultrasounds, MRIs, transient elastography scores, serum biomarker scores, and clinical notes for evidence of cirrhosis /stage 4 fibrosis. Based on the evidence, the trained site coordinators categorized patients as cirrhotic or noncirrhotic (Yes/No). All cross-referenced information in the dataset (e.g., labs, APRI, FIB-4, MELD, treatment type, duration, use of ribavirin) was reviewed to validate the accuracy of the cirrhosis categorization. When needed, sites were queried for additional information to support cirrhosis categorization (e.g., transient elastography scores). Adjudication of cases with inconsistent data was made by an experienced hepatologist (M.W.F.) who reviewed all available cross-referenced information or in less than .05% of cases, final adjudication was conducted by the site investigator who had access to the comprehensive medical record. These laboratory biomarkers and clinical data were explored as potential predictor variables.

#### Mental health and substance abuse history

Participants responded to five questions related to psychiatric history, two questions from the AUDIT[44] related to frequency and quantity of alcohol consumption, and two questions from the Substance Abuse Mental Illness Symptoms Screener (SAMISS) queried frequency of drug abuse in the past year, including use of nonprescription street drugs and prescription drugs [45]. Psychiatric questions queried (Yes/No) past and current psychiatric medication use, psychiatric diagnoses, psychiatric treatment, and inpatient psychiatric hospitalization.

### Statistical analysis strategy

#### Descriptive statistics

To characterize the study cohort, graphical and tabular descriptive statistical methods (means, standard deviations (SD), range) were used to visualize the data and describe the empirical distributions of the four PRO constructs: (1) Number of medical comorbidities; (2) Specific Symptom Clusters (PROMIS instruments); (3) Overall Symptom Burden (TMSAS); and (4) Functional Well-Being (HCV-PRO), as well as participant characteristics (sociodemographics, liver-related features, psychiatric and substance abuse history).

#### Bivariate associations

To help characterize relationships between each patient characteristic and each PRO, unadjusted point and interval estimates of correlation coefficients were tabulated along with unadjusted estimates of subgroup PRO means/medians. Pearson correlation coefficients were used for continuous-vs-continuous pairs of variables; point-biserial correlation coefficients were used for binary (e.g. gender, cirrhosis)-vs-continuous pairs; Spearman correlation coefficients were used for ordinal (e.g., cirrhosis status combined with MELD score)-vs-continuous pairs; and for continuous PRO measures and multi-level nominal categorical variables (e.g., race, employment), we used the square root of the unadjusted R^2^ for the general linear model of the PRO measure conditional on the categorical variable as a predictor. The square root of R^2^ is a non-directional absolute value (r).

#### Multivariable models of association

In the inferential investigation of patient characteristics that may be predictive of PROs, we implemented a cross-validation strategy. Participants were assigned by randomization to two groups: sample 1 or sample 2. Sample 1 (n = 700) was used for exploratory model-building analyses to generate a set of candidate predictor variables that might be associated with each outcome. Sample 2 (n = 900) was used to test and potentially validate the candidate models. Essentially, two identical studies were performed in which the second was used to validate predictor models generated by the first.

Based on documented and informal information from the data collection process, missing values of the outcome variables and the candidate predictor variables are presumed missing at random or missing completely at random. Complete-case analyses (omitting patients with missing data) suffers from selection bias and loss of precision. Therefore, missing values for the predictor variables were addressed via multiple imputation. Patients having a missing value for an outcome variable were omitted from that regression analysis. A multivariate multiple imputation algorithm (SAS procedure MI) was used to generate 40 completed copies of the dataset for the multivariable analyses. Each statistical regression model of interest was fitted to all 40 datasets. The 40 sets of results were combined (SAS procedure MIANALYZE) to produce the final results shown for each multivariable regression model.

The candidate predictor variables for model-building based in Sample 1 are found in Table 1 and include: (1) sociodemographics; (2) liver-related clinical and laboratory markers; and (3) mental health and substance abuse variables. Some of the laboratory markers were transformed to log_10_ scale. In using Sample 1 to select candidate multivariable models for each PRO, we first examined models accounting for sociodemographics, then we looked at the additional contribution of liver lab and clinical variables, and finally psychiatric and substance abuse variables were included. Model-building with Sample 1 relied on stepwise variable selection algorithms or use LASSO (least absolute shrinkage and selection operator) variable-selection algorithms. Having completed all exploratory analyses and model development with Sample 1 (n = 700), we then used the data from Sample 2 (n = 900) for validation. Candidate predictor variables in models based on Sample 2 were considered “*validated*” if their regression coefficients were statistically significant at level *α = 0*.*01*.

**Table 1:** Patients characteristics (n = 1600).

Characteristic	n (%) unless specified
**Sociodemographics**	
**Age,** years (mean (SD), range)	57 (11), 22-85
**Sex**	
Male	886 (55)
Female	714 (45)
**Race**	
Black	519 (33)
White	974 (61)
Other	101 (6)
Ethnicity	
Non-White Hispanic	66 (4)
**Marital Status**	
Single	570 (36)
Divorced, Separated, Widowed	442 (28)
Married or Domestic Partner	573 (36)
**Education Level**	
≤ High school diploma or equivalent	863 (55)
> High school	720 (45)
**Annual Household Income**	
≤ $40K	1164 (75)
> $41K	398 (25)
**Employment**	
Working full or part time	549 (36)
Receiving or applying for disability	696 (45)
Unemployed	110 (7)
Retired/homemaker/student	191 (12)
**Insurance status**	
Private insurance	542 (37)
Public insurance Medicaid/Medicare	750 (51)
Uninsured or hospital assistance	177 (12)
**Liver Lab and Clinical Markers**	**Mean (SD), range**
**HCV RNA level (log**_**10**_**IU/ml)**	6 (1), 1-7
**AST (IU/L)**	70 (63), 10-1324
**ALT (IU/L)**	78 (69), 6-682
**Albumin (g/dL)**	4 (0.5), 1.6-5.1
**Total Bilirubin (mg/dL)**	1 (1.1), 0.4-21
**Platelets (10**^**3**^**/**μ**L)**	197 (79), 25-610
**Hemoglobin (g/dL)**	14 (2), 6-19
**Creatinine (mg/dL)**	1 (1) 0.6-14.9
**INR**	1 (0.2), 1-3.9
	**n (%)**
**Genotype**	
Genotype 1	1285 (81)
Genotype 2-6	293 (19)
**Cirrhosis status**	
Cirrhotic	764 (48)
Noncirrhotic	825 (52)
**MELD status in cirrhotics**	
MELD 6-11	543 (85)
MELD ≥ 12	94 (15)
**AST to Platelet Ratio Index (APRI)**	
APRI ≤ 2.0	1313 (86)
APRI > 2.0	210 (14)
**Fibrosis-4 (Fib-4)**	
FIB-4 ≤ 3.25	1088 (71)
FIB-4 > 3.25	435 (29)
**Psychiatric and Substance Use Markers**	**n (%)**
**History of psychiatric medication use**	803 (50)
If yes, currently taking psychiatric medications	487 (30)
**History of mental health diagnosis**	701 (44)
**History of mental health treatment**	654 (41)
**History of psychiatric hospitalization**	287 (18)
**Frequency of current alcohol consumption**	
Never	1020 (64)
Monthly	384 (24)
Weekly	195 (12)
**Number of alcoholic drinks on a drinking day**	
None	1001 (63)
1 or 2	385 (24)
3 to 6	177 (11)
7 or more	31 (2)
**Use of nonprescription street drugs in last year**	
Never	1277 (80)
Less than monthly	113 (7)
Monthly	57 (4)
Weekly	56 (3)
Daily / Almost daily	90 (6)
**Use of prescription drugs to get high or change the way you feel in last year**	
Never	1453 (91)
Less than monthly	61 (4)
Monthly	23 (1)
Weekly	19 (1)
Daily / Almost daily	39 (3)

Model-based analyses for the patient-reported ‘Number of Medical Comorbidities’ relied on generalized log-linear models representing a mean count of comorbid conditions as a function of patient characteristics. Similarly, multivariable analyses for TMSAS and HCV-PRO measures relied on generalized linear models. The PROMIS Fatigue and PROMIS Sleep Disturbance T-scores also were studied using generalized linear models because they exhibited symmetric discrete distributions.

In contrast, the other eight symptom-specific PROMIS T-scores exhibited semi-continuous empirical distributions; that is, the scores follow a discrete distribution with a clumping of values at the floor of each scale (patients who reported no symptom). Therefore, the analysis of each of these 8 PROMIS measures relied on a generalized linear model for zero-inflated Poisson distributions. To represent both the probability of having the symptom and the conditional mean score as functions of patient characteristics, the zero-inflated Poisson model specifies two simultaneous regression equations for the modified T-score: one for the distribution of non-zero values (patients who have the symptom) and one for the clump of values at zero (for patients without the symptom). The dependent variable was computed as (T-score **−** K) with K being the observed floor of the scale. The floor values (K) were: Depression score > 42; Anger score > 35; Anxiety score > 45; Cognitive Concerns > 25; Pain Interference > 45; Belly Pain > 30; Diarrhea > 32; Nausea/Vomiting > 37 (these floor values were also used for other analyses of dichotomized T-scores). In these analyses, we focused on identifying patient characteristics associated with the non-zero values (having the symptom) to identify patients at risk for high symptom burden.

For all the PROs, sensitivity analyses included variations on the type of model fitted, variations on the engine used for exploratory hypothesis generation (e.g. stepwise, LASSO, least-angle regression, model averaging) and variations on the assumptions and criteria used (e.g. link function, α-levels for entry and exit). For the PROMIS T-scores, sensitivity analyses included logistic regression for the dichotomized T-scores, cumulative logit model analysis for categorized T-scores (low, medium, high), and zero-inflated negative binomial model analysis. All statistical estimates were computed along with corresponding 95% confidence intervals. Statistical computations were performed using SAS System software version 9.4 (SAS Institute, Cary, NC). PROMIS T-scores were computed using R software, version 3.1.2 (2014 The R Foundation for Statistical Computing), and RStudio software, version 1.0.136 (RStudio Inc.).

## Results

**Fig 1:**
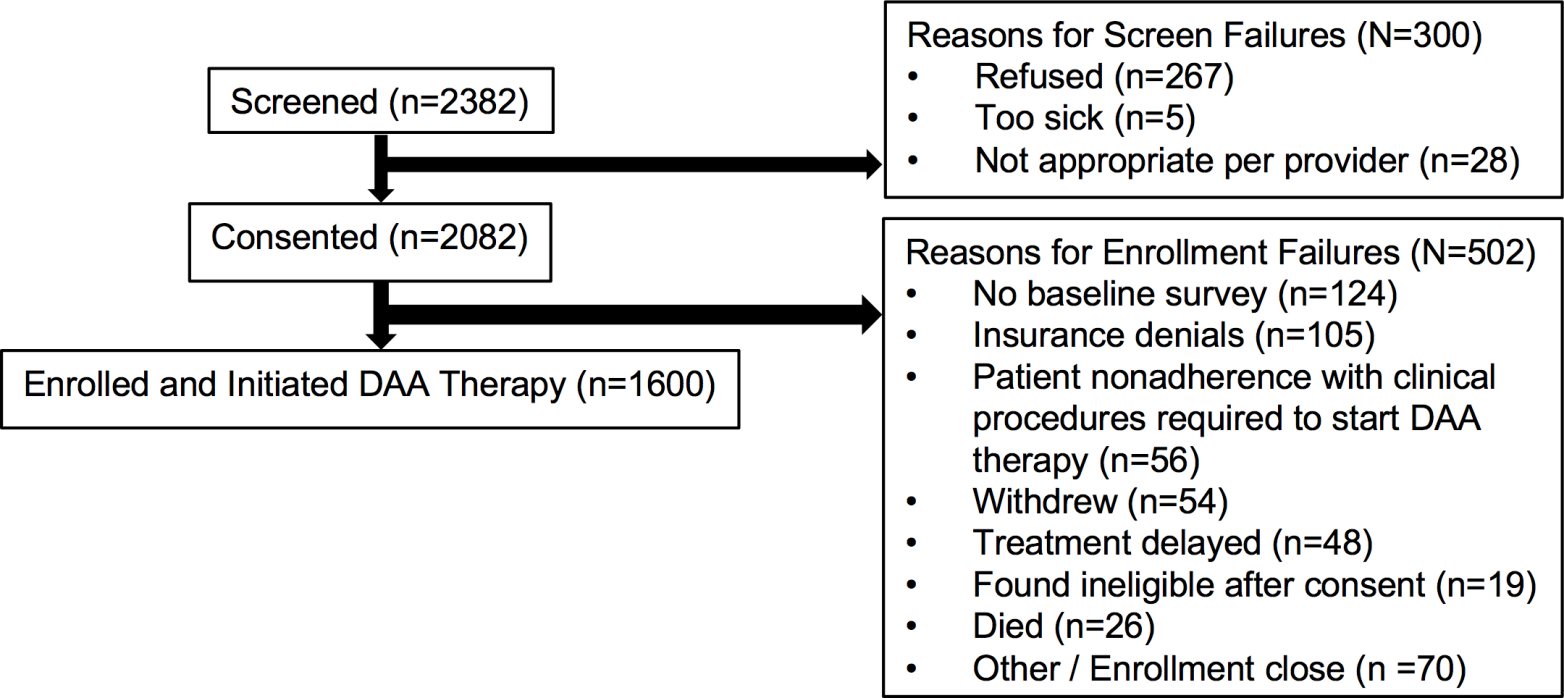
Study flowchart.

The study flowchart, which includes the number of patients screened, consented, and enrolled in PROP UP, is displayed in Fig 1. Of the nearly 2,400 patients screened, 87% consented to participate in the study. Of the 2082 consented patients, the main reasons patients were not enrolled included incomplete baseline surveys at the time that treatment was initiated (6%); insurance payer denials of DAA therapy (5%); patient lack of follow through with clinical requirements to start DAA therapy (e.g., completion of paperwork, urine toxicology screens) (3%); and verbal withdrawal from the study after consent (3%).

To explore the potential for selection bias that could affect generalizability of the study results, we compared the 1600 enrolled patients with 757 patients who were screened or consented, but not enrolled, on age, sex, and race. Compared to the 1600 patients enrolled, the 757 screen/enrollment failures were similar in age (55 years versus 57 years), sex (61% male versus 55%), and race (59% White versus 61%; 29% Black versus 33%).

### Patient characteristics

Baseline patient characteristics are detailed in Table 1. Notably, 39% of patients self-identified as Black/African American (n = 519) or Other Race (n = 101), which was comprised of ‘other’ (n = 41), bi-racial/multi-racial (n = 32), American Indian (n = 17), Asian (n = 6), ‘not reported’ (n = 6) and native Hawaiian/Pacific Islander (n = 5). Annual household income was low (less or equal to $40,000) in 75% of patients. Around 45% of patients reported being recipients of disability benefits or in the process of applying for disability benefits, 36% were working a part-time or full-time job, and 7% were unemployed, out of work or looking for work. Fifty-one percent of patients had public health insurance (Medicare or Medicaid), 12% were uninsured, and 37% had private insurance or private plus Medicare.

### Number of medical comorbidities and association with patient characteristics

In addition to liver-related problems, patients reported the presence of current and past comorbid conditions, 97% of these conditions was endorsed by at least one participant. The 10 most frequently reported comorbidities are listed in Table 2. Patients endorsed a mean of four comorbidities (range: 0–15) (S2 Table). Less than 10% (n = 154) reported no medical comorbidities, thus the majority had at least one medical condition other than chronic HCV. Notably, many of these comorbid conditions could represent EHDs.

**Table 2:** Top 10 patient reported medical comorbidities.

Medical Comorbidities	Current Symptoms n (%)	Past Symptoms n (%)	Combined Symptoms^a^ n (%)
Joint Pain	804 (50)	92 (6)	863 (55)
High Blood Pressure	790 (49)	122 (8)	887 (56)
Muscle Aches	686 (43)	78 (5)	740 (47)
Vision Loss or Problems	509 (32)	73 (5)	560 (36)
Sleep Disorder/Insomnia	494 (31)	63 (4)	535 (34)
Chronic Pain Disorder/Orthopedic	473 (30)	75 (5)	517 (33)
Diabetes/High Sugar Levels	327 (20)	46 (3)	369 (23)
Asthma/COPD	282 (18)	61 (4)	330 (21)
Hearing Loss or Problems	267 (17)	34 (2)	290 (18)
High Cholesterol	254 (16)	89 (6)	333 (21)

^a^ Each participant only counted once in combined column.

As shown in Table 3, a greater number of medical conditions correlated with sociodemographics (.20 ≤ r ≤ .40; being older, low income, public health insurance, disability), liver-related markers (.17 ≤ r ≤ .18; MELD ≥ 12, low albumin, low hemoglobin, high creatinine), and mental health markers (15 ≤ r ≤ .24; psychiatric medication use, diagnosis, treatment, inpatient hospitalization). Correlations with alcohol and substance abuse were lower (0 ≤ r ≤ .09).

**Table 3:** Patient characteristic subgroup means and correlations with PRO measures.

PATIENT CHARACTERISTICS	Number of Comorbid Conditions	TMSAS	Depression	Anger	Anxiety	Cognitive Concerns	Pain Interference	Fatigue	Sleep Disturbance	Belly Pain	Diarrhea	Nausea/ Vomiting	HCV-PRO
	Mean	Mean	Mean	Mean	Mean	Mean	Mean	Mean	Mean	Mean	Mean	Mean	Mean
	*r*	*r*	*r*	*r*	*r*	*r*	*r*	*r*	*r*	*r*	*r*	*r*	*r*
**SOCIODEMOGRAPHICS**													
**Age (Mean = 57 years old)**	***0*.*23***	***-0*.*05***	***-0*.*13***	***-0*.*19***	***-0*.*16***	***-0*.*13***	***0*.*00***	***-0*.*13***	***-0*.*09***	***-0*.*09***	***0*.*03***	***-0*.*10***	***0*.*12***
**Sex**	***0*.*07***	***0*.*15***	***0*.*10***	***0*.*06***	***0*.*06***	***0*.*08***	***0*.*04***	***0*.*15***	***0*.*05***	***0*.*08***	***0*.*05***	***0*.*06***	***0*.*05***
Male	3.8	0.5	48.4	48.3	49.8	32.3	52.6	50.3	51.2	35.1	35.2	41.2	72.6
Female	4.2	0.7	50.5	49.7	51.1	33.8	53.5	53.6	52.5	37.3	36.1	42.2	70.5
**Race**	***0*.*06***	***0*.*12***	***0*.*13***	***0*.*15***	***0*.*13***	***0*.*13***	***0*.*04***	***0*.*18***	***0*.*13***	***0*.*10***	***0*.*03***	***0*.*11***	***0*.*14***
Black	4.1	0.5	47.4	46.4	48.6	31.2	52.3	49.0	49.7	34.3	35.3	40.5	76.0
White	3.9	0.6	50.2	50.1	51.1	33.8	53.2	53.0	52.7	36.8	35.7	42.0	69.9
Other	4.5	0.7	51.3	50.6	53.1	33.8	53.7	53.9	53.2	38.5	36.3	43.8	66.8
**Marital Status**	***0*.*11***	***0*.*13***	***0*.*13***	***0*.*03***	***0*.*12***	***0*.*07***	***0*.*14***	***0*.*10***	***0*.*07***	***0*.*10***	***0*.*03***	***0*.*10***	***0*.*13***
Single	4.0	0.6	49.5	48.7	50.6	33.1	53.0	51.0	51.6	36.1	35.6	41.6	71.2
Divorced, Separated, Widowed	4.5	0.7	51.1	49.4	52.0	33.8	55.1	53.5	53.0	38.0	36.0	42.9	67.8
Married, domestic partner	3.6	0.5	47.8	48.6	48.9	32.3	51.3	51.2	51.0	34.7	35.3	40.7	75.0
**Education**	***0*.*10***	***0*.*11***	***0*.*08***	***0*.*08***	***0*.*08***	***0*.*08***	***0*.*11***	***0*.*01***	***0*.*09***	***0*.*08***	***0*.*05***	***0*.*11***	***0*.*08***
≤ High school diploma	4.3	0.6	50.0	49.6	51.1	33.7	54.0	51.9	52.7	37.1	36.0	42.5	70.1
> High school diploma	3.7	0.5	48.4	47.9	49.5	32.2	51.7	51.6	50.6	34.9	35.1	40.7	73.6
**Annual Income**	***0*.*20***	***0*.*20***	***0*.*18***	***0*.*09***	***0*.*15***	***0*.*13***	***0*.*25***	***0*.*13***	***0*.*13***	***0*.*16***	***0*.*07***	***0*.*15***	***0*.*22***
≤ $40K	4.3	0.6	50.4	49.4	51.3	33.7	54.6	52.6	52.6	37.3	35.9	42.4	68.8
> $41K	3.0	0.4	46.1	47.2	47.7	30.9	48.2	49.3	49.1	32.5	34.5	39.5	80.3
**Employment**	***0*.*40***	***0*.*36***	***0*.*28***	***0*.*19***	***0*.*27***	***0*.*27***	***0*.*42***	***0*.*24***	***0*.*26***	***0*.*27***	***0*.*17***	***0*.*26***	***0*.*39***
Working full or part time	2.7	0.4	46.0	47.1	47.7	30.5	48.1	49.2	48.7	32.3	33.8	39.3	80.4
Receiving or applying for disability	5.2	0.8	52.2	51.0	53.1	35.5	57.9	54.3	54.7	39.6	37.0	44.0	62.6
Unemployed	3.3	0.6	51.6	50.0	52.7	33.9	52.8	53.4	53.7	38.3	36.4	42.1	69.0
Retired/homemaker/student	3.7	0.4	46.7	45.8	46.8	30.2	49.0	48.6	48.5	33.3	34.8	39.7	81.0
**Health insurance**	**0.32**	***0*.*24***	***0*.*19***	***0*.*13***	***0*.*18***	***0*.*17***	***0*.*31***	***0*.*17***	***0*.*20***	***0*.*20***	***0*.*11***	***0*.*22***	***0*.*27***
Private insurance	2.8	0.4	46.7	47.1	48.0	31.0	48.5	49.4	48.9	32.8	34.3	39.3	79.6
Public insurance Medicaid/Medicare	4.9	0.7	50.5	49.5	51.5	34.2	55.7	53.3	53.3	38.2	36.3	43.0	66.7
Uninsured or hospital assistance	3.9	0.6	51.7	51.5	53.0	34.3	54.8	52.7	53.8	38.0	36.2	43.4	68.1
**LIVER LAB & CLINICAL MARKERS**													
HCV RNA viral load (log_10_IU/ml) (Mean = 6)	***0*.*03***	***0*.*04***	***0*.*04***	***0*.*02***	***0*.*02***	***0*.*03***	***0*.*05***	***0*.*03***	***0*.*01***	***0*.*02***	***0*.*04***	***0*.*01***	***-0*.*04***
AST (IU/L) (Mean = 70)	***0*.*00***	***0*.*06***	***0*.*03***	***0*.*04***	***-0*.*02***	***0*.*02***	***-0*.*01***	***0*.*03***	***0*.*08***	***0*.*04***	***0*.*03***	***0*.*01***	***-0*.*05***
ALT (IU/L) (Mean = 78)	***-0*.*07***	***0*.*00***	***0*.*02***	***0*.*08***	***0*.*00***	***0*.*01***	***-0*.*04***	***0*.*02***	***0*.*06***	***-0*.*01***	***-0*.*03***	***-0*.*03***	***-0*.*05***
Albumin (g/dL) (Mean = 4)	***-0*.*18***	***-0*.*16***	***-0*.*05***	***-0*.*02***	***-0*.*01***	***-0*.*03***	***-0*.*11***	***-0*.*09***	***-0*.*08***	***-0*.*12***	***-0*.*14***	***-0*.*08***	***0*.*09***
Total Bilirubin (mg/dL (Mean = 1)	***0*.*01***	***0*.*04***	***-0*.*01***	***-0*.*03***	***-0*.*03***	***-0*.*02***	***-0*.*02***	***-0*.*01***	***0*.*05***	***0*.*01***	***0*.*02***	***0*.*03***	***-0*.*01***
Platelets (10^3^/μL) (Mean = 197)	***-0*.*05***	***-0*.*06***	***-0*.*04***	***-0*.*06***	***0*.*00***	***-0*.*03***	***0*.*01***	***-0*.*02***	***-0*.*05***	***-0*.*04***	***-0*.*07***	***0*.*00***	***0*.*06***
Hemoglobin (g/dL) (Mean = 14)	***-0*.*18***	***-0*.*12***	***0*.*00***	***0*.*01***	***0*.*02***	***-0*.*01***	***-0*.*07***	***-0*.*07***	***-0*.*01***	***-0*.*07***	***-0*.*11***	***-0*.*06***	***0*.*00***
Creatinine (mg/dL) (Mean = 1)	***0*.*13***	***0*.*02***	***0*.*00***	***0*.*00***	***-0*.*02***	***-0*.*02***	***0*.*05***	***0*.*02***	***0*.*03***	***0*.*00***	***0*.*04***	***0*.*06***	***-0*.*01***
INR (Mean = 1)	***0*.*10***	***0*.*07***	***0*.*02***	***0*.*02***	***0*.*01***	***0*.*03***	***0*.*04***	***0*.*04***	***0*.*06***	***0*.*10***	***0*.*11***	***0*.*06***	***-0*.*06***
**Cirrhosis status**	***0*.*07***	***0*.*05***	***0*.*03***	***0*.*01***	***0*.*02***	***0*.*05***	***0*.*02***	***0*.*03***	***0*.*05***	***0*.*05***	***0*.*03***	***0*.*02***	***0*.*05***
No	3.8	0.5	48.9	49.0	50.6	32.7	52.7	51.4	51.3	35.4	35.3	41.5	72.8
Yes	4.2	0.6	49.7	48.7	50.2	33.3	53.2	52.1	52.3	36.7	35.9	41.9	70.6
**If cirrhotic, MELD score**	***0*.*09***	***0*.*09***	***0*.*05***	***0*.*00***	***0*.*02***	***0*.*05***	***0*.*04***	***0*.*05***	***0*.*06***	***0*.*06***	***0*.*05***	***0*.*06***	***0*.*07***
MELD 6–11	4.0	0.6	49.4	48.3	49.8	33.1	53.3	51.9	51.9	36.3	35.1	41.5	71.4
MELD ≥ 12	5.3	0.8	51.4	50.8	51.9	34.8	55.1	54.9	55.2	40.1	40.2	44.9	64.5
**AST to Platelet Ratio Index (APRI)**	***0*.*02***	***0*.*09***	***0*.*07***	***0*.*06***	***0*.*01***	***0*.*06***	***0*.*00***	***0*.*08***	***0*.*10***	***0*.*07***	***0*.*11***	***0*.*05***	***0*.*09***
APRI ≤ 2.0	4.0	0.6	49.0	38.6	50.3	32.7	53.0	51.4	51.3	35.7	35.2	41.5	72.6
APRI > 2.0	4.2	0.7	51.0	50.6	50.8	34.4	53.1	54.1	54.8	38.3	38.0	42.8	66.5
**Fibrosis-4**	***0*.*06***	***0*.*07***	***0*.*02***	***0*.*00***	***0*.*02***	***0*.*01***	***0*.*01***	***0*.*02***	***0*.*03***	***0*.*04***	***0*.*07***	***0*.*03***	***0*.*03***
FIB-4 ≤ 3.25	3.9	0.5	49.1	48.9	50.5	32.9	52.9	51.6	51.6	35.7	35.2	41.5	72.2
FIB-4 > 3.25	4.3	0.6	49.7	48.8	50.1	33.1	53.3	52.2	52.4	36.9	36.5	42.1	70.7
**PSYCHIATRIC AND SUBSTANCE USE MARKERS**													
**History of psychiatric medication use**	**0.21**	**0.35**	**0.38**	**0.30**	**0.36**	**0.33**	**0.27**	**0.30**	**0.24**	**0.20**	**0.10**	**0.19**	**0.38**
No	3.4	0.4	45.3	45.5	46.7	30.0	50.0	48.5	49.0	33.5	34.7	40.0	80.0
Yes	4.6	0.7	53.2	52.2	54.0	36.0	55.9	55.0	54.5	38.6	36.4	43.2	63.2
**Currently on psychiatric medications**	***0*.*12***	***0*.*23***	***0*.*22***	***0*.*12***	***0*.*19***	***0*.*19***	***0*.*14***	***0*.*16***	***0*.*04***	***0*.*15***	***0*.*10***	***0*.*13***	***0*.*17***
No	4.2	0.6	50.3	50.5	51.5	33.8	53.9	53.0	53.9	36.0	35.2	41.8	67.9
Yes	4.9	0.8	55.1	53.3	55.6	37.4	57.1	56.3	54.8	40.3	37.1	44.2	60.0
**History of mental health diagnosis**	***0*.*24***	***0*.*33***	***0*.*36***	***0*.*28***	***0*.*35***	***0*.*33***	***0*.*27***	***0*.*29***	***0*.*20***	***0*.*17***	***0*.*11***	***0*.*20***	***0*.*37***
No	3.4	0.4	46.0	46.0	47.3	30.3	50.3	49.0	49.7	34.1	34.7	40.1	79.0
Yes	4.8	0.8	53.6	52.5	54.4	36.4	56.3	55.3	54.3	38.6	36.7	43.6	62.1
**History of mental health treatment or services**	***0*.*16***	***0*.*27***	***0*.*30***	***0*.*25***	***0*.*30***	***0*.*29***	***0*.*17***	***0*.*24***	***0*.*15***	***0*.*14***	***0*.*09***	***0*.*18***	***0*.*29***
No	3.6	0.5	46.7	46.6	47.8	30.8	51.4	49.5	50.3	34.6	34.9	40.3	77.2
Yes	4.5	0.7	53.0	52.2	54.0	36.1	55.1	54.9	53.7	38.2	36.5	43.4	64.0
**History of psychiatric hospitalization**	***0*.*15***	***0*.*24***	***0*.*24***	***0*.*21***	***0*.*26***	***0*.*25***	***0*.*14***	***0*.*19***	***0*.*11***	***0*.*15***	***0*.*10***	***0*.*18***	***0*.*24***
No	3.8	0.5	48.1	47.8	49.2	31.9	52.2	50.8	51.1	35.2	35.2	40.9	74.2
Yes	4.9	0.8	54.7	53.9	55.9	37.9	56.4	56.2	54.5	40.1	37.5	44.8	60.0
**Frequency of current alcohol consumption**	***0*.*08***	***0*.*10***	***0*.*03***	***0*.*03***	***0*.*09***	***0*.*09***	***0*.*09***	***0*.*06***	***0*.*05***	***0*.*11***	***0*.*02***	***0*.*04***	***0*.*09***
Never	4.2	0.6	49.6	49.2	51.1	33.6	53.7	52.3	52.2	37.1	35.7	41.9	70.2
Weekly to monthly consumption	3.7	0.5	48.9	48.4	49.2	31.8	51.6	50.9	51.1	34.3	35.4	41.2	74.4
**Number of alcoholic drinks on a drinking day**	***0*.*09***	***0*.*10***	***0*.*03***	***0*.*03***	***0*.*09***	***0*.*09***	***0*.*10***	***0*.*06***	***0*.*05***	***0*.*12***	***0*.*02***	***0*.*04***	***0*.*09***
None	4.2	0.6	49.6	49.2	51.1	33.6	53.8	52.2	52.3	37.3	35.7	41.9	70.1
1+	3.7	0.5	48.9	48.5	49.3	31.9	51.6	51.0	51.0	34.1	35.3	41.2	74.2
**Use of nonprescription street drugs in last year**	***0*.*00***	***0*.*07***	***0*.*10***	***0*.*11***	***0*.*08***	***0*.*07***	***0*.*02***	***0*.*03***	***0*.*07***	***0*.*04***	***0*.*03***	***0*.*09***	***0*.*05***
Never	4.0	0.6	48.8	48.3	50.0	32.7	52.8	51.6	51.4	35.8	35.4	41.2	72.2
Daily to monthly	4.0	0.6	51.3	51.3	52.1	34.2	53.5	52.4	53.5	37.0	36.2	43.3	69.3.
**Use of prescription drugs to get high or change the way you feel in last year**	***0*.*05***	***0*.*12***	***0*.*14***	***0*.*15***	***0*.*13***	***0*.*14***	***0*.*07***	***0*.*11***	***0*.*11***	***0*.*08***	***0*.*07***	***0*.*15***	***0*.*12***
Never	4.0	0.6	48.8	48.3	50.0	32.6	52.7	51.4	51.4	35.8	35.4	41.2	72.5
Daily to monthly	4.5	0.8	54.2	54.4	54.5	36.9	55.6	55.7	55.8	39.2	37.4	45.8	63.1

Mean scores for all patient characteristic subgroups by PRO measures and correlation coefficients (***r***) as degree of relationship between the predictor variable and PRO measure. Correlations range from 0 (no association) to +/- 1.0 (perfect association).

Table 4 shows the results of the confirmatory multivariable analyses (n = 900) predicting *Number of Medical Comorbidities* and other PROs. The estimates are the predicted percent (%) increase in the mean score attributable to a specific patient characteristic. For example, a *higher Number of Medical Comorbidities* was associated with being older (2.7% higher for each additional year) and being disabled (53% higher).

**Table 4:** Validated predictors of PROs.

Entries are % increase in the mean score with 95% C.I.	Number of Medical Comorbidities	Overall Symptom Burden (TMSAS)	Functional Well-Being^a^ (HCV-PRO)	PROMIS Fatigue T-score	PROMIS Sleep Disturbance T-score
Older Age	2.7 [2.1, 3.4]				
Female		13 [5, 22]		6 [3, 9]	
White race				9 [6, 13]	
Other race				10 [4, 17]	
Unemployed		29 [12, 45]	-11 [-17, -4]		10 [4,16]
Disabled	53 [36, 71]	41 [31, 51]	-15 [-19, -11]	9 [5, 13]	10 [6, 13]
APRI > 2			-6 [-11, -2]		
Currently on Psych Meds		33 [19, 48]	-11 [-17, -6]	7 [2, 13]	8 [3, 13]
History of Psych Meds					7 [2, 12]

^a^ For the HCV-PRO, higher scores are better; estimates reflect the % decrease in HCV-PRO score (lower well-being).

### Specific symptom clusters and association with patient characteristics

**Fig 2:**
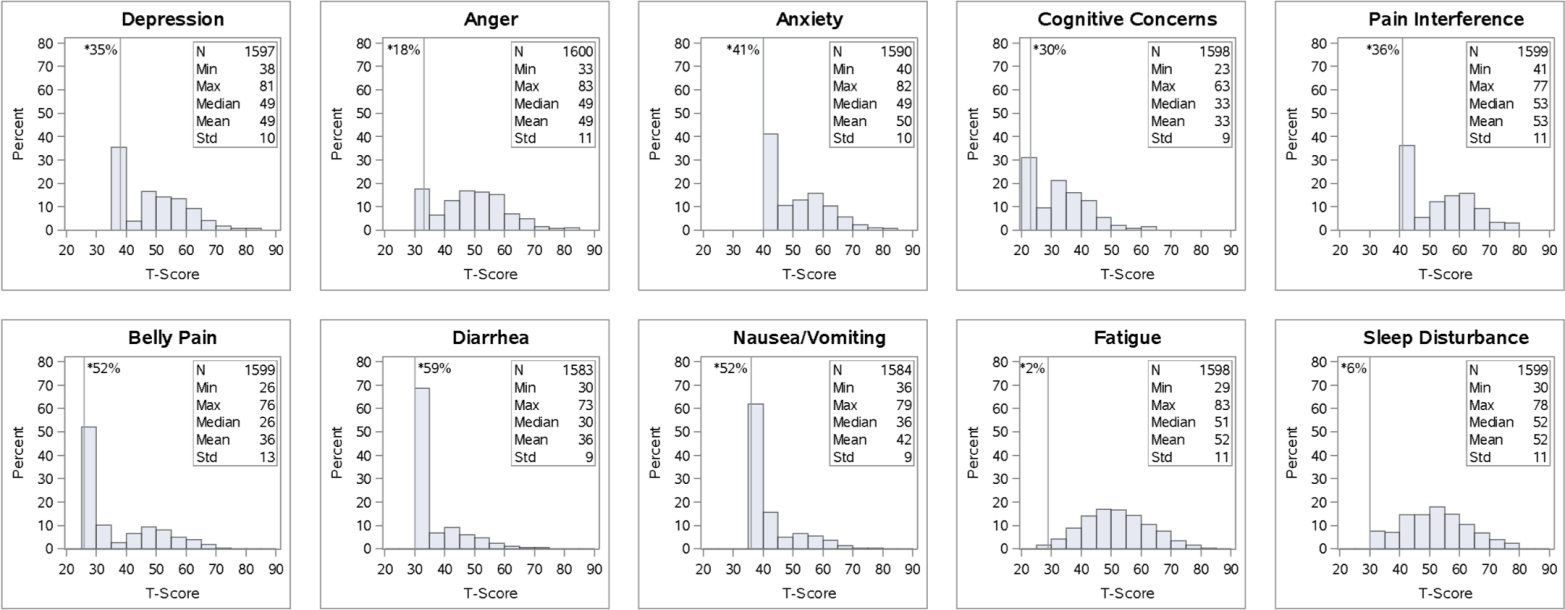
Histograms of PROMIS symptom T-scores. *The vertical line in each histogram shows the proportion of patients reporting no symptoms or responses at the minimum score.

Fig 2 displays histograms for the T-scores for each of the PROMIS symptoms. Eight PROMIS measures showed a bimodal distribution with some patients reporting no symptoms while others reported mild to severe symptoms. T-scores over 55 are 1/2 standard deviation worse than the general US population and considered a clinically meaningful difference in other medical populations[34, 46–48].

#### Neuropsychiatric symptom cluster

Approximately 60-80% of patients endorsed symptoms in the neuropsychiatric symptom cluster (Fig 2). Bivariate correlations and unadjusted mean group differences are summarized in Table 3. Of the sociodemographic variables, the largest correlations with this symptom cluster were with employment (.19 ≤ r ≤ .28), health insurance (.13 ≤ r ≤ .19), and income (.09 ≤ r ≤ .18), such that disability, unemployment, low income, and public health insurance were associated with worse symptoms. Correlations between the neuropsychiatric symptom cluster and liver-related markers were quite small (0 ≤ r ≤ .07).

In multivariable models, severity of the neuropsychiatric symptom cluster was frequently associated with being disabled, unemployed, and use of psychiatric medications (Table 5). Patients who were disabled, unemployed, white, single and with past or current psychiatric medication use had 6% to 18% higher *depression* scores. *Anger severity* decreased with age and was 4% to 8% higher in patients who were disabled, unemployed, using psychiatric medications and abusing prescription drugs. *Anxiety severity* decreased with age and was 6% to 11% higher in patients who were disabled, of other race, uninsured, taking psychiatric medications, or had a history of psychiatric diagnosis. Severity in *cognitive concerns* was 4% to 9% higher in patients who were disabled, unemployed, on psychiatric medications, and history of psychiatric hospitalization.

**Table 5:** Validated predictors of T-score symptom severity.

Entries are % increase in the mean scorewith 95% C.I.	Depression	Anger	Anxiety	Cognitive Concerns	Pain	Belly Pain	Diarrhea	Nausea/ Vomiting
Younger Age		-.3 [-.4, -.2]	-.3 [-.4, -.1]					
Other Race			10 [5, 16]					
White Race	6 [3, 9]							
Low Income					5 [2, 8]			
Disabled	14 [10, 18]	7 [6, 9]	11 [7, 16]	7 [4, 9]	13 [11, 16]	18 [14, 21]	19 [15, 23]	23 [18, 28]
Unemployed	18 [13, 23]	5 [2, 8]		8 [3, 12]	8 [3, 13]	13 [9, 18]		22 [14, 30]
Retired				-7 [-12, -3]				11 [3, 19]
Uninsured			8 [2, 15]					
Single	5 [2, 8]							
Currently on Psych Meds	16 [12, 20]	8 [5, 10]	10 [6, 14]	9 [6, 13]	5 [1, 8]	11 [8, 15]	7 [2, 13]	
Hx of Psych Meds	8 [4, 12]	4 [2, 6]			5 [2, 8]	9 [6, 13]		
Prescription drug abuse		5 [3, 7]						14 [9, 18]
Hx of Psych diagnosis			6 [2, 10]			-5 [-8, -2]	-8 [-13, 3]	
Hx of Psych hospitalized				4 [1, 7]			7 [3, 12]	

#### Somatic symptom cluster

Almost all patients endorsed some level of fatigue and sleep disturbance, evidenced by the histograms in Fig 2 and 65% reported pain interference. Bivariate correlations and unadjusted mean differences between the somatic symptom cluster and patient characteristics are summarized in Table 3. In general, the largest correlations with the somatic symptom cluster were the sociodemographic and psychiatric variables. Patients who had lower income, were unemployed or disabled, or on public health insurance had worse somatic symptoms (.13 ≤ r ≤ .42) Patients with psychiatric markers were also observed to experience worse pain, sleep, and fatigue (.11 ≤ r ≤ .29). By contrast, correlations between the somatic symptom cluster and liver-related markers were small (.01 ≤ r ≤ .11).

Multivariable models demonstrated that *fatigue severity* was worse among patients who were female (6% higher), White or other race (9%-10% higher), disabled (9% higher) and those taking psychiatric medications (7% higher) (Table 4). *Sleep disturbance severity* was higher in patients who were unemployed (10% higher), disabled (11% higher), using psychiatric medications (7%-8% higher) (Table 4). Patients with lower income (5% higher), disabled (13% higher), unemployed (8% higher), with psychiatric medication use (5% higher) reported more *severe pain interference* (Table 5). No liver-related markers were identified as validated predictors of the somatic symptom cluster.

#### Gastrointestinal symptom cluster

Approximately half of the cohort reported symptoms in the GI cluster, including abdominal pain (50%), nausea/vomiting (50%), and diarrhea (40%) (Fig 2). Bivariate correlations between the GI symptom cluster and patient characteristics are summarized in Table 3. Correlations were smaller with the GI cluster than with other symptom clusters. In general, the sociodemographic variables had the largest correlations with the GI cluster (.07 ≤ r ≤ .27), followed by correlations with the psychiatric markers (.10 ≤ r ≤ .20), and liver-related markers (0 ≤ r ≤ .11).

In multivariable models, *nausea/vomiting severity* was worse in patients who were disabled, unemployed or abusing prescription medications (14% to 23% higher) (Table 5). *Abdominal pain severity* was worse in patients who were disabled, unemployed, and with psychiatric diagnosis and psychiatric medication use (5% to 18% higher). *Diarrhea severity* was worse in patients who were disabled, taking psychiatric medications, and with a history of psychiatric diagnosis and hospitalization (7% -19% higher).

### Overall symptom burden and associations with patient characteristics

On the MSAS, individual symptoms reported by at least 25% of participants are listed in Table 6 (S3 Table). The median TMSAS score was 0.4 (mean = 0.6, SD = 0.5, range: 0–3.1). The most common symptoms were lack of energy, pain, and difficulty sleeping. These symptoms were also the most severe and frequent.

**Table 6:** MSAS symptoms reported by at least 25% of patients.

Symptoms	% Endorsed	% Severity^a^	% Frequency^b^	% Distressing^c^
Lack of energy	60	50	39	43
Pain	52	46	37	42
Difficulty sleeping	48	42	34	35
Worrying	43	33	23	28
Numbness/tingling in hands/feet	40	31	23	26
Feeling drowsy	37	28	17	21
Dry mouth	34	24	18	17
Feeling irritable	32	22	12	20
Difficulty concentrating	31	20	13	20
Feeling nervous	31	24	14	21
Feeling sad	29	22	13	20
Cough	27	17	12	12
Feeling bloated	27	20	13	17
Shortness of breath	25	19	11	17
Itching	25	18	11	15

Symptom severity, frequency and distress are reported as percentages of those who endorsed the symptom presence.

^a^ Severity ranges from slight, moderate, severe, very severe; data shown is % reporting moderate, severe, very severe symptoms.

^b^ Frequency ranges from rarely, occasionally, frequently, almost constantly; data shown is % reported frequently or almost constantly.

^c^ Distress or bothersome ranges from not at all, a little bit, somewhat, quite a bit, very much; data shown is % reporting symptoms as somewhat, quite a bit, or very much distressing.

As shown in Table 3, higher overall symptom burden (higher TMSAS scores) was correlated with sociodemographics (.15 ≤ r ≤ .36; being female; lower income; disabled; uninsured; having public health insurance), lab biomarkers (.12 ≤ r ≤ .16; lower albumin, lower hemoglobin) and psychiatric markers (.23 ≤ r ≤ .35). Correlations with alcohol and substance abuse were lower (0 ≤ r ≤ .12). In multivariable regressions, *symptom burden* was higher in patients who were female (13% higher), disabled (41% higher), unemployed (29% higher), and taking psychiatric medications (33% higher) (Table 4).

### HCV-specific functional well-being

The mean HCV-PRO total score was 72 (median = 77, SD = 22; range = 0-100), a score consistent with a previous Phase II clinical trial where patients had a mean baseline HCV-PRO of 78[40]. As shown in Table 3, patients with lower functional well-being tended to be younger; other race; lower income, disabled, unemployed, or on public insurance. Low functional well-being was also correlated with the mental health markers (.24 ≤ r ≤ .38). In contrast, the correlations between HCV-PRO scores and liver-related markers were quite small, with the strongest correlations being with albumin, APRI >2, and MELD ≥12 (.07 ≤ r ≤ .09). In multivariable regression models, *functional well-being* was lower in patients who had worse liver disease (APRI >2) (6% lower), were unemployed (11% lower), disabled (15% lower), and taking psychiatric medications (11% lower).

### Sensitivity analyses

Sensitivity analyses and diagnostics were used to evaluate the robustness of the reported main results in Tables 4 and 5 to reasonable perturbations of the statistical methods and assumptions used. These auxiliary analyses were used to guide our level of trust in the main results. The various sensitivity analyses produced results similar to the main results. For example: **(1)** For Table 5, similar results were obtained when using a zero-inflated negative binomial (ZINB) model instead of the zero-inflated Poisson (ZIP) model, albeit with wider confidence intervals as expected. The two approaches identified the same predictor variables but ZINB classified fewer as “validated”. In terms of the Akaiki Information Criterion, the ZINB fit was slightly better for some PRO measures. **(2)** For Table 4, similar results were obtained when using a generalized linear model with identity link function instead of the Poisson model. **(3)** Results in Tables 4 and 5 were supported when we explored inclusion or exclusion of selected candidate predictor variables. **(4)** Finally, the results in Tables 4 and 5 relied on multiple imputation (MI) of missing values for patient characteristics using a two-step approach: Markov-chain-Monte-Carlo estimation followed by use of parametric regressions. Closely similar versions of Tables 4 and 5 were obtained when relying on an alternative MI method in which the second step was a nonparametric propensity scores method.

## Discussion

With recent advances in the treatment of HCV using highly potent DAAs and resultant changes to demographics of patients initiating DAA treatment, a contemporary and comprehensive assessment of the symptom burden of HCV-infected individuals in a real world setting, outside of carefully selected clinical trials populations, was needed. Towards this end, we conducted an in-depth analysis of patient-reported symptom burden including HCV-associated specific symptoms, medical comorbidities and functional well-being in a large multicenter cohort initiating DAA therapy. We also took the opportunity to thoroughly describe the cohort with regard to several sociodemographics, liver-related features, and mental health and drug and alcohol use parameters of the current treatment population. Through a rigorous analytical approach, we identified and validated key patient characteristics associated with each PRO. Our analysis has three key findings. First, many patients with chronic HCV, although not all, had a large number of medical comorbidities and high symptom burden, with the most common being fatigue, sleep disturbance, pain interference and neuropsychiatric symptoms. Second, most PROs were strongly associated with just a handful of patient characteristics; namely, disability, unemployment and current use of psychiatric medications. Other predictor variables for each PRO were identified, but with lower frequency and strength of association. Third, laboratory biomarkers and clinical markers of liver disease severity were not strong predictors of most PROs. These findings will lay the groundwork for subsequent longitudinal investigations of symptoms and comorbid conditions that may change over the course of DAA therapy and after viral eradication.

This study provides extensive sociodemographic information regarding the chronic HCV population currently being treated in the US. The study cohort was 61% White and 39% non-White (33% identified as Black/African-American, 6% identified as Other Race), in contrast to industry-sponsored clinical trials that under-represent minority populations (i.e., majority are >80% White (range: 66%-97%)[49–51]. The vast majority of patients initiating treatment had low educational attainment (not exceeding high school) and low household income (less or equal to $40,000 per year), well below the average income cited in the 2016 Census survey[52]. Over 50% were recipients of disability benefits, applying for disability benefits, or unemployed and looking for work. The majority was receiving public health insurance or was uninsured. Employment status, including disability and unemployment, as well as low income and lack of insurance were common patient features associated with high symptom burden and comorbidities, consistent with the larger literature on social and economic determinants of poor health in other medical populations). Recognizing the impact of SDoH on the health and treatment outcomes of the HCV population has both clinical and health policy implications. While the emphasis has been on viral eradication to improve liver-related outcomes, our results highlight the need to acknowledge the high burden of concomitant chronic health conditions among the chronic HCV population. In fact, HCV therapy initiation presents a window of opportunity for patients to re-engage with the healthcare system and address other health conditions. Improving the overall health of the HCV population will require improvements and innovation at multiple levels of healthcare including enhanced coordination of care between hepatologists and other subspecialists, multidisciplinary teams, co-location of mental health and addiction specialists, patient navigation, and innovative telehealth models [21, 53–63].

Nearly half of the cohort had cirrhosis and 12% had evidence of advanced cirrhosis (MELD ≥ 12). We suspect that state Medicaid restrictions during the enrollment period partially influenced variability of liver disease severity in this cohort by limiting access to treatment to patients with higher stages of fibrosis and denying treatment to those with minimal fibrosis[64, 65].

Psychiatric disorders were common comorbidities among HCV-infected individuals in the interferon-era[13, 66], and this trend continues. Almost half of the patients reported a psychiatric diagnosis (44%), utilizing mental health treatment or services (41%), or psychiatric medication use (50%), with 30% currently on psychiatric medications. Eighteen percent (n = 287) of the cohort reported a past history of inpatient psychiatric hospitalizations, a proxy for severe psychopathology. The prevalence of these mental health indicators far exceeds the prevalence in the general US population[67]. A total of 36% of patients were still using alcohol at time of treatment initiation and at least 20% had used street drugs and 9% reported using prescription medications in an abusive fashion in the previous year. While these data are alarming, it is likely that patients under-reported these behaviors due to social desirability and fear of treatment being rescinded. In this study, we attempted to mitigate under-reporting by highlighting the confidential nature of data collection and separation of research team and clinical staff. The high prevalence of psychiatric disorders and moderate drug and alcohol use has clinical relevance for all practitioners, as these patients will be at risk for long-term poor health outcomes, beyond liver disease.

The vast majority of patients suffer from multiple chronic health conditions. Patients reported up to 15 comorbid conditions and an average of four. A greater number of comorbidities was found for patients who are disabled and older. Many of the prevalent medical conditions could represent biologically plausible inflammatory conditions, or extrahepatic disorders (EHDs) related to HCV, such as diabetes[68, 69]. Recent literature suggests that EHDs are underestimated because they are non-specific, but may compromise overall health outcomes and result in an estimated economic burden of $1.5 billion per year[70]. The diagnostic guidelines published by the International Study Group of Extrahepatic Manifestations Related to HCV recommend evaluating all patients with HCV for potential EHDs to ensure that the entire spectrum of HCV-related disorders are identified and properly treated[4]. Indeed, patients with all stages of liver disease should be treated for HCV and not delayed until fibrosis advances, as studies indicate that clinical and economic burden of EHDs can be reduced through viral eradication[70, 71].

Like comorbidities, many, but not all patients reported experiencing specific symptoms. For each of the specific neuropsychiatric, somatic and GI symptoms, a proportion of patients did not experience that particular symptom (e.g., 50-60% had no GI symptoms), but the majority reported mild to severe symptoms. For instance, fatigue and sleep disturbance were ubiquitous problems in the entire cohort. With regard to the neuropsychiatric cluster (depression, anxiety, anger, cognitive concerns), about 60-80% endorsed mild to severe symptoms, with severe neuropsychiatric symptoms experienced by patients who were disabled, unemployed, and on psychiatric medications, among other factors. Over 60% of patients endorsed pain interference and 30% endorsed joint/muscle pain, which is consistent with previous reports of pain disorders in patients with chronic HCV[6, 15, 72–74]. Over 90% of patients reporting some level of sleep disturbance; 31% reported a sleep disorder. Estimates from the literature suggest that up to 60% of patients with chronic HCV may have sleep problems, including restless leg syndrome, although confounding effects of other comorbidities has been difficult to tease apart[6, 75]. Disability, unemployment, and psychiatric medication use were the prominent predictors of the pain, sleep interference, and fatigue cluster. Finally, 40-50% of patients endorsed GI symptoms (nausea/vomiting, abdominal pain, diarrhea) and similarly, disability, unemployment and psychiatric markers including psychiatric medication use were associated with more severe GI symptoms.

A comprehensive analysis of symptom prevalence in the chronic HCV population has not been conducted since the work of Lang et al. in 2006[6]; therefore, this study provides a contemporary perspective of the most prevalent, severe, and distressing symptoms in the chronic HCV population. The majority of symptoms endorsed by at least 20% of patients in this cohort overlap considerably with Lang et al.’s findings, validating our results. We also identified new symptoms (e.g., numbness and tingling in hands and feet, dry mouth, cough, feeling bloated, shortness of breath, and lack of sexual interest) that were prevalent and require further investigation. This information can help clinicians identify and better care for these patients and observe changes in symptoms after viral eradication.

The functional well-being of the current cohort was comparable to that obtained from the original HCV-PRO analysis embedded in a Phase II clinical trial[40]. Items endorsed on the HCV-PRO most often included “Having Hepatitis C was very stressful to me”, “I had difficulty sleeping or slept too much”, and “I needed to pace myself to finish what I had planned”. HCV-specific functional well-being was worse in patients with advanced liver disease (APRI > 2), and those disabled, unemployed and on psychiatric medications. The HCV-PRO is a contemporary disease-specific instrument developed with rigorous methods and we would recommend it for use in future HCV investigations; unfortunately, no other studies have been published to date, therefore cross-study comparisons are unable to be conducted.

Not surprisingly, we found that laboratory biomarkers and markers of liver disease severity were not strong predictors of the PROs investigated in this study. We observed differences in the unadjusted means between high and low MELD subgroups by 2 to 7 points across the PROs, reflecting possible clinically significant differences in patients with advanced cirrhosis on symptoms such as sleep disturbance and the GI symptom cluster. However, MELD was not a strong predictor in confirmatory models. There were small differences between patients with high versus low APRI, FIB-4, albumin and creatinine, but only APRI was validated as a predictor of functional well-being in multivariable models. Our findings are consistent with a large body of HrQOL literature from the interferon treatment era that found negligible associations between liver disease parameters and PROs once other variables were accounted for[15, 76–81]. Most of these studies have found psychiatric and/or medical conditions to be the strongest predictors of PROs in multivariate models, especially depression and pain-related conditions[15, 80–82]. Many clinical researchers have speculated that HCV exerts a deleterious effect on PROs through medical and psychiatric comorbidities, which could represent underlying HCV-induced EHDs [15, 80, 81]. These speculations require further investigation.

Limitations of this study should be noted, particularly regarding the generalizability of these results. Our findings may not apply to other subpopulations or clinical settings, including younger people who inject drugs, persons receiving medication-assisted treatment for opioid use disorders with methadone or buprenorphine/naloxone, Veterans, and those incarcerated. These subgroups may represent the primary treatment groups in years to come if the opioid epidemic is not controlled[83]. We found no substantial differences between patients enrolled and not enrolled in this study, therefore we can presume generalizability to similar patients treated in similar settings. Social desirability and response bias issues could have occurred, especially in response to alcohol and drug use questions. However, it’s important to appreciate the necessity of capturing data directly from patients when they are uniquely positioned to assess an outcome (i.e., pain), as research shows that clinicians tend to under-report the frequency and severity of patients’ symptoms[84]. Finally, minimal clinical data were extracted from medical records, thus we are unable to verify patient-reporting of medical comorbidities, psychiatric medications and other clinical information.

Future research should build upon our observations to directly inform clinical knowledge and decision-making for various stakeholders (patients, clinicians, policy makers). There is a critical need to tease apart and elucidate the causal pathways that may influence health outcomes in the HCV population (i.e., psychiatric illness, EHDs, number of medical comorbidities, liver disease, symptoms); this may be achieved through sophisticated path analyses and structural equation modeling[85]. We identified a new predictor of worse PROs, namely employment status, which should be included in future predictive models of HCV-related PROs. Health services research is needed to examine the most effective models to integrate healthcare for liver disease, mental health, and addiction[21, 55–57, 60–63]. More studies derived from real world clinical settings, outside of clinical trials, need to examine changes in overall symptom burden and specific symptom clusters over time, during DAA therapy, after virologic cure, and during long-term follow-up. Comparisons between the DAA regimens and how they affect PROs would provide stakeholders with information to guide treatment decision-making. Importantly, it remains unclear if overall symptom burden lessens after virologic cure or if only *specific symptom clusters* improve. Therefore, the exploratory work herein to identify and validate the most robust predictors of PROs is fundamental to future PRO research in chronic HCV.

Our findings have a number of strengths and highlight clinical relevance for multiple stakeholders. This study is unique and notably the largest investigation of PROs in the current population of HCV patients seeking DAA therapy outside of clinical trials. We have identified some of the most challenging sociodemographic features, common comorbidities, potentially EHDs, and troublesome symptoms experienced by HCV-infected individuals. More black Americans have been recruited than any previously conducted clinical trial and thus provided an opportunity for a robust evaluation. Interestingly, Black patients reported the lowest levels of symptom burden and neuropsychiatric symptoms. Consistent with many prior studies, we found that laboratory biomarkers and liver disease severity were not helpful in identifying patients with diminished PROs, such as HrQOL, when other variables were accounted for[80–82]. It is also noteworthy that PROP UP is a highly patient-centered study. Patients affected by HCV were engaged since its inception and helped to identify the study outcomes and select PRO measures to ensure the study findings were salient and meaningful to people with HCV. Finally, given the large sample size, we were able to conduct cross-validation analyses to rigorously identify and confirm predictors, essentially conducting two separate identical studies, and then followed up with sensitivity analyses to confirm that perturbations in modeling, missing data, and assumptions did not alter our findings. These methods serve to instill a higher level of trust in the conclusions drawn from this study. Consequently, we hope the comprehensive data provided herein serves as reference guide for future investigations of PROs in the chronic HCV patient population.

## Supporting information

S1 Table Full names of each subsite's local approving IRBs. (DOCX)

S2 Table Complete list of self-reported medical comorbidities. ^a^ Each participant only counted once in combined column.
(DOCX)

S3 Table Complete list of MSAS symptoms. Symptom severity, frequency and distress are reported as percentages of those who endorsed the symptom presence. ^a^ Severity ranges from slight, moderate, severe, very severe; data shown is % reporting moderate, severe, very severe symptoms. ^b^ Frequency ranges from rarely, occasionally, frequently, almost constantly; data shown is % reported frequently or almost constantly. ^c^ Distress or bothersome ranges from not at all, a little bit, somewhat, quite a bit, very much; data shown is % reporting symptoms as somewhat, quite a bit, or very much distressing.
(DOCX)

## Abbreviations

ALT: alanine aminotransferase test
AST: aspartate aminotransferase tes
APRI: AST to platelet ratio index
AUDIT: Alcohol Use Disorders Identification Test
CI: Confidence interval
DAA: Direct acting antiviral
EHDs: extrahepatic disorders
FIB-4: Fibrosis-4 Index for Liver Fibrosis
GI: Gastrointestinal
HCV: hepatitis C virus
HrQOL: health-related quality of life
Hx: history
IFN: Interferon
INR: international normalized ratio
IRB: Institutional Review Board
LASSO: Least absolute shrinkage and selection operator
MELD: Model for End-Stage Liver Disease
MSAS: Memorial Symptom Assessment Scale
PCORI: Patient-Centered Outcomes Research Institute
PRO: patient-reported outcome
PROMIS: Patient-Reported Outcome Measurement Information System
PROP UP: The Patient-Reported Outcomes Project of HCV-TARGET
RBV: Ribavirin
SD: Standard deviation
SDoH: social determinants of health
TMSAS: Total Memorial Symptom Assessment Scale
Tx: treatment
