# Supplementary material for: A comprehensive assessment of patient reported symptom burden, medical comorbidities, and functional well being in patients initiating direct acting antiviral therapy for chronic hepatitis C: Results from a large US multi-center observational study
Source: PLoS One. 2018 Aug 1;13(8):e0196908. doi: 10.1371/journal.pone.0196908 (PMC6070182; doi:10.1371/journal.pone.0196908)
Supplement: S1 Table — (DOCX) [file pone.0196908.s001.docx]

**Supporting Information**

**S1 Table: Full names of each subsite's local approving IRBs**

1. University of North Carolina at Chapel Hill

The UNC Office of Human Research Ethics and Institutional Review Board

1. University of Florida, Gainesville

The University of Florida Institutional Review Board

1. Rush University Medical Center

Rush Institutional Review Board

1. Saint Louis University

Saint Louis University Institutional Review Board

1. University of California at Davis

University of California, Davis Institutional Review Board Administration

1. University of Michigan

University of Michigan Medical School Institutional Review Board

1. University of Pennsylvania

University of Pennsylvania Institutional Review Board

1. Virginia Commonwealth University

VCU Office of Research and Innovation

1. Yale University

Yale Human Investigation Committee

1. Asheville Gastroenterology Associates

UNC Office of Human Research Ethics and Institutional Review Board

1. Wilmington Gastroenterology Associates

UNC Office of Human Research Ethics and Institutional Review Board
