# Supplementary material for: A comprehensive assessment of patient reported symptom burden, medical comorbidities, and functional well being in patients initiating direct acting antiviral therapy for chronic hepatitis C: Results from a large US multi-center observational study
Source: PLoS One. 2018 Aug 1;13(8):e0196908. doi: 10.1371/journal.pone.0196908 (PMC6070182; doi:10.1371/journal.pone.0196908)
Supplement: S2 Table — a Each participant only counted once in combined column. (DOCX) [file pone.0196908.s002.docx]

**Supporting Information**

**S2 Table. Complete list of self-reported medical comorbidities.**

| Medical Comorbidities | Current Symptom N (%) | Past Symptoms N (%) | Combined Symptoms^a^ N (%) |
| --- | --- | --- | --- |
| Joint Pain | 804 (50) | 92 (6) | 863 (55) |
| High Blood Pressure | 790 (49) | 122 (8) | 887 (56) |
| Muscle Aches | 686 (43) | 78 (5) | 740 (47) |
| Vision Loss or Problems | 509 (32) | 73 (5) | 560 (36) |
| Sleep Disorder/Insomnia | 494 (31) | 63 (4) | 535 (34) |
| Chronic Pain Disorder/Orthopedic | 473 (30) | 75 (5) | 517 (33) |
| Diabetes/High Sugar Levels | 327 (20) | 46 (3) | 369 (23) |
| Asthma/COPD | 282 (18) | 61 (4) | 330 (21) |
| Hearing Loss or Problems | 267 (17) | 34 (2) | 290 (18) |
| High Cholesterol | 254 (16) | 89 (6) | 333 (21) |
| Digestive Disorder | 223 (14) | 58 (4) | 274 (17) |
| Skin Disorder/Psoriasis | 154 (10) | 46 (3) | 192 (12) |
| Heart Disease | 146 (9) | 56 (4) | 194 (12) |
| Thyroid Problems | 139 (9) | 48 (3) | 184 (12) |
| Genital/Urinary Disorder | 111 (7) | 55 (3) | 159 (10) |
| Kidney Disease | 95 (6) | 48 (3) | 141 (9) |
| Lung/Pulmonary Disease | 97 (6) | 34 (2) | 126 (8) |
| Fibromyalgia | 93 (6) | 16 (1) | 105 (7) |
| Cancer, any kind | 87 (5) | 171 (11) | 252 (16) |
| HIV Infection | 57 (4) | 4 (0) | 58 (4) |
| Blood/Coagulation/Clotting Disorder | 63 (4) | 41 (3) | 103 (7) |
| Stomach Ulcers | 69 (4) | 109 (7) | 172 (11) |
| Hepatitis B | 54 (3) | 79 (5) | 128 (8) |
| Liver transplant | 30 (2) | 25 (2) | 51 (3) |
| IBD/Crohn's Disease/Colitis | 34 (2) | 24 (2) | 56 (4) |
| Kidney transplant | 16 (1) | 23 (1) | 39 (2) |
| Stroke/TIA | 23 (1) | 104 (7) | 126 (8) |
| Seizures | 19 (1) | 73 (5) | 90 (6) |
| Porphyria | 9 (1) | 0 (0) | 9 (1) |
| Heart Transplant | 3 (0) | 2 (0) | 3 (0) |

^a^ Each participant only counted once in combined column.
