# Supplementary material for: A comprehensive assessment of patient reported symptom burden, medical comorbidities, and functional well being in patients initiating direct acting antiviral therapy for chronic hepatitis C: Results from a large US multi-center observational study
Source: PLoS One. 2018 Aug 1;13(8):e0196908. doi: 10.1371/journal.pone.0196908 (PMC6070182; doi:10.1371/journal.pone.0196908)
Supplement: S3 Table — Symptom severity, frequency and distress are reported as percentages of those who endorsed the symptom presence. a Severity ranges from slight, moderate, severe, very severe; data shown is % reporting moderate, severe, very severe symptoms. b Frequency ranges from rarely, occasionally, frequently, almost constantly; data shown is % reported frequently or almost constantly. c Distress or bothersome ranges from not at all, a little bit, somewhat, quite a bit, very much; data shown is % reporting symptoms as somewhat, quite a bit, or very much distressing. (DOCX) [file pone.0196908.s003.docx]

**Supporting Information**

**S3 Table. Complete List of MSAS Symptoms.**

| Symptoms | % Endorsed | % Severity^a^ | % Frequency^b^ | % Distressing^c^ |
| --- | --- | --- | --- | --- |
| Lack of energy | 60 | 50 | 39 | 43 |
| Pain | 52 | 46 | 37 | 42 |
| Difficulty sleeping | 48 | 42 | 34 | 35 |
| Worrying | 43 | 33 | 23 | 28 |
| Numbness/tingling in hands/feet | 40 | 31 | 23 | 26 |
| Feeling drowsy | 37 | 28 | 17 | 21 |
| Dry mouth | 34 | 24 | 18 | 17 |
| Feeling irritable | 32 | 22 | 12 | 20 |
| Difficulty concentrating | 31 | 20 | 13 | 20 |
| Feeling nervous | 31 | 24 | 14 | 21 |
| Feeling sad | 29 | 22 | 13 | 20 |
| Cough | 27 | 17 | 12 | 12 |
| Feeling bloated | 27 | 20 | 13 | 17 |
| Shortness of breath | 25 | 19 | 11 | 17 |
| Itching | 25 | 18 | 11 | 15 |
| Sweats | 23 | 18 | 12 | 13 |
| Problems with sexual interest or activity | 21 | 18 | 15 | 15 |
| Lack of appetite | 20 | 15 | 10 | 10 |
| Nausea | 19 | 14 | 7 | 12 |
| Dizziness | 19 | 12 | 4 | 10 |
| Constipation | 19 | 13 | 0 | 10 |
| Swelling of arms or legs | 18 | 12 | 0 | 10 |
| Problems with urination | 17 | 14 | 10 | 12 |
| Diarrhea | 14 | 10 | 5 | 8 |
| Weight loss | 13 | 6 | 0 | 4 |
| Changes in skin | 12 | 8 | 0 | 7 |
| I don't look like myself | 11 | 8 | 0 | 7 |
| Hair loss | 9 | 5 | 0 | 5 |
| Change in the way food tastes | 8 | 4 | 0 | 4 |
| Difficulty swallowing | 7 | 5 | 3 | 5 |
| Vomiting | 5 | 4 | 1 | 3 |
| Mouth sores | 4 | 2 | 0 | 2 |

Symptom severity, frequency and distress are reported as percentages of those who endorsed the symptom presence. ^a^ Severity ranges from slight, moderate, severe, very severe; data shown is % reporting moderate, severe, very severe symptoms. ^b^ Frequency ranges from rarely, occasionally, frequently, almost constantly; data shown is % reported frequently or almost constantly. ^c^ Distress or bothersome ranges from not at all, a little bit, somewhat, quite a bit, very much; data shown is % reporting symptoms as somewhat, quite a bit, or very much distressing.
